# Evaluation of the gut microbiome and sex hormones in postmenopausal women with newly diagnosed hormone receptor-positive breast cancer versus healthy women: a prospective case-control study

**DOI:** 10.1007/s00432-025-06338-z

**Published:** 2025-10-04

**Authors:** Maryann Kwa, Grant Hussey, Yelena Novik, Adrian A. Franke, Angelina Volkova, Karina Flores, Martin J. Blaser, James Speyer, Ruth Oratz, Marleen Meyers, Komal Jhaveri, Ezeddin Fadel, Adriana Heguy, Jonas Schluter, Kelly V. Ruggles, Sylvia Adams

**Affiliations:** 1 Laura and Isaac Perlmutter Cancer Center, New York University Grossman School of Medicine, New York, NY USA; 2 Institute for Systems Genetics, New York University Grossman School of Medicine, New York, NY USA; 3 Division of Precision Medicine, Department of Medicine, New York University Grossman School of Medicine, New York, NY USA; 4 Analytical Biochemistry Shared Resource, University of Hawaii Cancer Center, Honolulu, HI USA; 5 Institute for Computational Medicine, Department of Microbiology, New York University Grossman School of Medicine, New York, NY USA; 6 Center for Advanced Biotechnology and Medicine, Rutgers University, Piscataway, NJ USA; 7 Breast Medicine Service and Early Drug Development, Memorial Sloan Kettering Cancer Center, New York, NY USA; 8 Center for Biospecimen Research and Development, New York University Grossman School of Medicine, New York, NY USA; 9 Genome Technology Center, Department of Pathology, New York University Grossman School of Medicine, New York, NY USA; 10 Department of Microbiology, New York University Grossman School of Medicine, New York, NY USA

**Keywords:** Breast cancer, Microbiome, Estrogen, Progesterone

## Abstract

**Purpose:**

The functional composition and diversity of the gut microbiome may affect breast cancer risk by modulation of systemic sex hormones. Gut bacteria with β-glucuronidase enzymatic activity may deconjugate estrogens, leading to increased estrogen reabsorption into the circulation thereby increasing breast cancer risk. We investigated the relationship between the gut bacterial microbiome and endogenous estrogens and related sex hormones in women with hormone receptor-positive breast cancer compared to healthy control women. The goal was to determine if the estrobolome (i.e., bacteria capable of modulating the body’s circulated estrogen levels) was altered in those with breast cancer compared with controls.

**Methods:**

In this prospective case–control study, postmenopausal women (*n* = 46) with newly diagnosed stage I-III estrogen and/or progesterone receptor-positive breast cancer were compared with healthy postmenopausal female controls (*n* = 22). Bacterial composition of the gut microbiome was analyzed by 16S rRNA gene sequencing from fecal specimens. Plasma and urine sex hormones were quantified using high-performance liquid chromatography/mass spectrometry.

**Results:**

We found evidence that some β-glucuronidase positive bacteria were enriched in the breast cancer patients compared to healthy controls, whereas abundances of some β-glucuronidase negative bacteria were reduced. There was also a wide distribution of prevalence of β-glucuronidase positive taxa in both breast cancer subjects and healthy controls, as well as higher probability of breast cancer subjects having higher average β-glucuronidase levels. Significant differences were found in endogenous progesterone levels between the breast cancer patients and healthy controls.

**Conclusion:**

This pilot study showed differences in the gut microbiome and endogenous progesterone levels among postmenopausal women with hormone receptor-positive breast cancer compared with healthy controls. These interesting findings may have implications for breast cancer risk and prevention and warrant further exploration.

**Supplementary Information:**

The online version contains supplementary material available at 10.1007/s00432-025-06338-z.

## Background

There has been growing interest in the functional role of the gut microbiome in malignancy. The human gut microbiome exerts both local and distant effects involving hormonal intermediates, metabolites, and immunologic pathways (Belkaid and Hand 2014; Maynard et al. 2012). Interactions between the human host and microbes have the potential to influence carcinogenesis through mechanisms such as chronic inflammation, metabolism, induction of genotoxic responses, and alteration of the microenvironment (Shapira et al. 2013; Hullar and Fu 2014). This may be mediated by the microbial ecosystem as a whole or by specific microbes, as in the case with bacterium, *Helicobacter pylori*, which is associated with an increased risk of stomach adenocarcinoma. We postulated that certain sex hormone-conjugate hydrolyzing gut bacteria microbiota could contribute to breast cancer risk by increasing circulating sex hormone levels.

In the United States, breast cancer affects one in eight women and is the second leading cause of cancer-related deaths in females (Siegel et al. 2021). The most common type of breast cancer is estrogen and/or progesterone receptor-positive (i.e., hormone receptor-positive), which comprises approximately seventy percent of patients, with the majority occurring in postmenopausal women (Kohler et al. 2015; Anderson et al. 2011). Epidemiologic studies have demonstrated a significant association between endogenous estrogen levels and risk for hormone receptor-positive breast cancer in postmenopausal women (Key et al. 2002; Fuhrman et al. 2014), and the combination of exogeneous estrogens with progesterone also increased risk in the Women’s Health Initiative trials (Chlebowski et al. 2015). On the other hand, the role of endogenous progesterone in breast cancer carcinogenesis is less well defined (Trabert et al. 2020a, b).

The ‘estrobolome’ is the aggregate of intestinal bacteria capable of metabolizing estrogens (Plottel and Blaser 2011). Estrogens are primarily produced by the ovaries in premenopausal women and by the adrenal glands and adipose tissue in postmenopausal women. They circulate in the bloodstream in free or protein-bound form and first undergo metabolism primarily in the liver, where they are conjugated (sulfation and glucuronidation), oxidized, reduced, and/or methylated (Zhu et al. 2006; Raftogianis et al. 2000). The water-soluble conjugated estrogens are excreted from the body through the kidneys into urine or via bile into the feces (Sandberg and Slaunwhite 1957). However, some of the conjugated estrogens excreted in bile can be deconjugated by resident bacterial taxa in the gut with β-glucuronidase enzymatic activity, subsequently leading to estrogen reabsorption into the circulation (Cole et al. 1985; Gloux et al. 2011; Dabek et al. 2008; McIntosh et al. 2012). Modulation of estrogen homeostasis through the enterohepatic circulation by the gut microbiome can differ amongst individuals (Adlercreutz et al. 1984; Goldin et al. 1982; Flores et al. 2012). It is important to investigate the composition of the gut estrobolome in breast cancer, as a potential target for therapeutic or preventative interventions such as probiotics, antibiotics, and dietary modifications.

We hypothesized that the gut microbiome is different in women with hormone receptor-positive breast cancer compared with healthy control women. The bacterial composition of the estrobolome may be affected by host factors such as age, ethnicity, and body mass index (BMI), as well as lifestyle and dietary habits (Mariat et al. 2009; Modi et al. 2014; Hullar, Burnett-Hartman, and Lampe 2014). We designed a prospective case–control study with a carefully defined patient population and assessments to investigate the relationship between the gut bacterial microbiome with β-glucuronidase activity and endogenous sex hormones. We postulated that the gut microbiome in postmenopausal women with breast cancer is enriched in bacterial taxa with β-glucuronidase activity compared to control women, and differences in endogenous estrogens and related sex hormones are associated with variability in gut microbial diversity between the women with breast cancer and those without.

## Methods

### Breast cancer and healthy control subjects

Postmenopausal women with newly diagnosed histologically-confirmed breast cancer that was estrogen receptor (ER) positive and/or progesterone receptor (PR) positive and human epidermal growth factor receptor 2 (HER2) negative with resected stage I to III disease were eligible, prior to adjuvant endocrine therapy. For healthy controls, postmenopausal women without a history of malignancy were eligible. Women were defined as postmenopausal if they were at least 60 years of age, had undergone bilateral oophorectomy, or were younger than 60 years of age with cessation of regular menses for at least twelve consecutive months and plasma levels of estradiol and follicle-stimulating hormone in the postmenopausal range. Exclusion criteria included medical illnesses with potential suppressive or activating impact on immune and bowel function as determined by the treating investigator, systemic antibiotic or probiotic use within six months, use of hormone-replacement therapy within the past twelve months, or a history of gastric or intestinal surgery.

### Study design

This was a prospective single institution case–control pilot study. Postmenopausal women with breast cancer were recruited from breast medical oncology practices at the NYU Langone Health’s Perlmutter Cancer Center (PCC) during routine office visits with their oncologists to discuss and/or initiate adjuvant endocrine therapy with an aromatase inhibitor. Postmenopausal women without breast cancer (controls) were recruited from three subject pools: (a) relatives and/or friends of patients; (b) women undergoing routine mammographic screening at PCC’s facilities; and (c) female faculty or staff members. All subjects with breast cancer were planned to receive adjuvant endocrine therapy with an aromatase inhibitor (i.e., anastrozole, letrozole, or exemestane). Enrolled breast cancer subjects provided serial plasma, stool, and urine samples at baseline (prior to initiation of endocrine therapy for the breast cancer patients), and then at 1, 3, 6, and 12 months while on endocrine therapy. Samples were collected at identical time points for the control subjects, of which none received endocrine therapy while enrolled on study. Analyses are reported on the baseline samples in this publication. The study was approved by the NYU School of Medicine Institutional Review Board (IRB). Written informed consent was obtained from all subjects prior to enrollment in the study. Subjects who completed the study received a one hour-massage at the NYU Integrative Health Center in appreciation for their participation.

### Sample and data collection

Sample collection (plasma, urine, and stool) followed standardized procedures. After providing informed consent, study subjects were provided with a specimen collection kit with instructions for collecting a fecal sample at home, and materials to transport the specimens to NYU PCC. Fecal specimens were collected in four 20-mL screw top Sarstedt tubes, including two preloaded with 5 mL RNAlater (QIAGEN) and two with 5 mL sterile PBS. After specimen collection, the fecal tubes were secured, and two frozen gel packs were added. Urine was collected in a screw-top container, without preservative in the clinic. Blood samples were obtained by venipuncture in the clinic and collected in heparin containing vacutainer tubes. All specimens were transported to the NYU biorepository, where they were processed and stored at − 80 °C. The urine and plasma specimens were shipped on dry ice in batches to the laboratory (A.F.) at University of Hawaii and stored at − 80 °C until analysis.

Subjects in both groups also completed a questionnaire at baseline requesting information about age, height and weight, ethnicity, medical history of immune or gastrointestinal disorders, medication use including antibiotics within past the six months, diet, alcohol use, and smoking history (Supplementary Fig. 1). Follow-up questionnaires were completed at each subsequent time point inquiring about any changes in the above. Subjects were also followed for development of breast cancer recurrence or new malignancies.

### Fecal microbiome analysis

The bacterial composition of the gut microbiome was assessed using 16S ribosomal RNA gene sequencing of subject fecal samples. FASTQ files were preprocessed with QIIME2 (v. 2018.11) (Bolyen et al. 2019). Following the data input, sequences underwent an error correction step with *qiime dada2 denoise-paired* (parameters: –p-trunc-len-f 0, –p-trunc-len-r 0, –p-trim-left-f 20, –p-trim-left-r 20) command. We then assigned taxonomy to the sequences by training a Naïve Bayes classifier on the V4 region of the 16S rRNA gene with *qiime feature-classifier fit-classifier-I-bayes command* based on the GreenGenes database (v 13_8).

Following taxonomic assignment, the feature abundance table was rarefied to a sequencing depth of 20,000 reads per sample to normalize for differences in sequencing depth and to reduce bias introduced by uneven sampling effort. After rarefaction, taxonomic abundances were collapsed at both the genus and species levels, aggregating operational taxonomic units (OTUs) based on their assigned taxonomy. This approach allowed for consistent comparisons of microbial community composition across samples at broader taxonomic ranks, while maintaining resolution where available.

### Sex hormone analysis

The 11 most predominant steroidal estrogens in women, namely estrone (E1), estradiol (E2), 2-hydroxyestrone (2-OHE1), 2-hydroxyestradiol (2-OHE2), 2-methoxyestrone (2-MeOE1), 2-hydroxy-3-O-methylestrone (2OH-3MeO-E1), 4-hydroxyestrone (4-OHE1), 4- hydroxyestradiol (4-OHE2), 16α-hydroxyestrone (16α-OHE1), 16-ketoestradiol (16keto-E2), and estriol (E3) (Eliassen et al. 2009) as well as progesterone, and testosterone were measured from plasma and urine by our validated orbitrap liquid chromatography-mass spectrometry (LCMS) (model Q-Exactive, Thermo Scientific, Waltham, MA) assay, as described in detail previously (Li and Franke 2015).

In brief, plasma or urine were mixed with ascorbic acid as preservative and deuterated or 13C labeled analytes as internal standards followed by enzymatic hydrolysis with beta-glucuronidase and sulfatase for total analyte levels (conjugated plus unconjugated analytes) or without hydrolysis for unconjugated analytes followed by extraction with methyl tertiary butyl ether. The dried extract was derivatized with 1-methylimidazole sulfonyl chloride in sodium bicarbonate followed by analysis of the tagged analytes with high-resolution accurate-mass mass spectrometry in positive mode after electrospray ionization using monoisotopic protonated analyte masses 5 ppm to account for measurement inaccuracies as detailed previously (Li and Franke 2015). Urinary creatinine levels were determined using a clinical autoanalyzer (Roche**-**Cobas MiraPlus CC) and a kit from Randox Laboratories (cat. No. CR 510, Crumlin, UK) based on the Jaffé reaction with a lower limit of quantitation of < 15 µM. In this study, we found inter- and intra assay cv values of 0.8% at 187 µM. Urinary concentrations were adjusted for creatinine levels to account for differences in urine volume and concentration of the collected urine samples.

Circulating sex hormone binding globulin (SHBG) levels were measured with a well validated enzyme linked immunosorbent assay kit from R&D Systems, Inc. (Minneapolis, MN, kit# DSHBG0B, lot# P248238) following exactly the manufacturer’s protocol.

### Statistical analysis

All downstream microbiome analyses, including assessments of α-diversity, β-diversity, and taxonomic composition, were conducted using R (version 3.5.2) with appropriate microbiome and statistical packages. α-diversity metrics such as Shannon and Simpson indices were calculated to evaluate species richness and evenness, while β-diversity was assessed using distance metrics such as Bray–Curtis and visualized through principal coordinates analysis (PCoA). To identify bacterial taxa that significantly differed between breast cancer patients and healthy controls, we employed LEfSe (Linear Discriminant Analysis Effect Size) as described by Segata et al. (2011). The analysis was conducted using an alpha value of 0.05 and an LDA score threshold of 2.0 to identify taxa most likely to explain group-level differences. Differences in measured analyte levels between breast cancer cases and healthy controls were assessed using an unpaired two-sample t-test assuming equal variance, performed in Microsoft Excel (version 16.54). To investigate associations between urinary estrogen levels and microbial taxa, we calculated Spearman rank correlation coefficients between estrogen concentration and the relative abundance of each bacterial taxon. OTUs were grouped at the species level when taxonomic resolution allowed, or otherwise at the genus level, resulting in a total of 202 taxa considered. To reduce noise from low-abundance taxa, correlation analyses were restricted to those present at a relative abundance greater than 0.001% in at least two patient samples (n = 102 taxa). P-values were computed for each correlation to assess statistical significance. All correlation analyses were conducted in Python (version 3.8.10) using the scipy library (version 1.5.3).

## Results

### Study participants

A total of 60 breast cancer patients (BC) and 25 healthy participants (HP) enrolled in the study and provided plasma, urine, and stool samples. Fourteen breast cancer patients and 3 control subjects withdrew study consent prior to completion of serial sample collection over one year, and the analyses are therefore performed on 46 and 22 subjects, respectively. Subject demographic data and lifestyle factors are shown in Table 1 (*n* = 68). The breast cancer patients had a mean age of 64 (range 45–84 years), and the controls had a mean age of 58 (range 51–68). The majority of subjects were Caucasian (63–64% in both groups). African American patients comprised 15% of the breast cancer patients and 14% of the controls, and Asian patients made up 11% and 14%, respectively. In both groups, 9% of subject were Hispanic. For the breast cancer patients, the mean BMI was 26.3 kg/m^2^ (range 18.0–37.1), and for the controls, the mean BMI was 25.6 kg/m^2^ (range 18.9–38.7).

**Table 1:** Demographic and lifestyle factors for breast cancer cases and controls (*n* = 68)

	Breast Cancer Cases (*n* = 46)	Controls (*n* = 22)	*p*-value
Mean age (range)	64 (45–84)	58 (51–68)	0.009*
Race (%)			
Caucasian	29 (63%)	14 (64%)	0.97
African American	7 (15%)	3 (14%)	0.87
Hispanic	4 (9%)	2 (9%)	0.96
Asian	5 (11%)	3 (14%)	0.76
Native Hawaiian or Other	1 (2%)	0 (0%)	0.49
Mean BMI [kg/m^2^] (range)	26.3 (18.0–37.1)	25.6 (18.9–38.7)	0.59
Smoking (%)			
Yes	1 (2%)	2 (9%)	0.20
No	45 (98%)	20 (91%)	
Diet (%)			
Vegetarian	4 (9%)	4 (18%)	0.29
Probiotic Use	11 (24%)	5 (23%)	0.92
Alcohol Use (%)	20 (43%)	12 (55%)	0.53
Number drinks/week (range)	1–21	0.5–7	

BMI, body mass index; kg, kilogram; m, meter

The majority of subjects in both groups did not smoke (98% in the breast cancer group and 91% in the control group). About 44% of breast cancer patients and 55% of controls reported drinking alcohol, although there were large variations in the amount consumed (number of drinks per week ranged from 1 to 21 for the breast cancer patients and 0.5–7 for the controls). Regarding diet, 9% of the breast cancer patients were vegetarian versus 18% in the controls. A similar percentage of patients in both groups used probiotics (24% in the breast cancer patients and 23% in the controls).

### Breast cancer patients and tumor characteristics

Clinical and tumor characteristics of the breast cancer patients (*n* = 46) are shown in Table 2. They had an ECOG performance status of 0 (52%) or 1 (48%). Invasive ductal carcinoma was the most common tumor histology at 85%, with the other 15% of tumors being invasive lobular carcinoma. The majority of tumors were grade 2 (moderately differentiated) at 63%, followed by grade 3 (poorly differentiated) at 22%, and grade 1 (well differentiated) at 15%. Seventy percent of patients had stage 1 disease, 28% had stage 2, and 2% has stage 3. All tumors (100%) were ER-positive and 89% were PR-positive. After surgery, four patients received chemotherapy and sixteen patients underwent radiation therapy to the breast prior to enrollment.

**Table 2:** Clinical and tumor characteristics of breast cancer cases (*n* = 46)

Characteristic	Number (%)
ECOG PS	
0	24 (52%)
1	22 (48%)
Tumor Histology	
Invasive Ductal Carcinoma (IDC)	39 (85%)
Invasive Lobular Carcinoma (ILC)	7 (15%)
Tumor Grade	
1 (well differentiated)	7 (15%)
2 (moderately differentiated)	29 (63%)
3 (poor differentiated)	10 (22%)
Pathologic Staging	
T1N0	31 (67%)
T2N0	9 (20%)
T1N1	1 (2%)
T2N1	4 (9%)
T3N1	1 (2%)
Overall Stage	
I	32 (70%)
II	13 (28%)
III	1 (2%)
Tumor Biomarkers	
ER positive	46 (100%)
PR positive	41 (89%)
HER2 positive	1 (2%)
Radiation Received Prior to Enrollment	16 (35%)
Chemotherapy Received Prior to Enrollment	4 (9%)

ECOG, Eastern Cooperative Oncology Group; PS, performance status; T, tumor; N, nodal; ER, estrogen receptor; PR, progesterone receptor; HER2, human epidermal growth factor receptor 2

### New malignancies

Three of the breast cancer patients were later diagnosed with another malignancy after completion of longitudinal sample collection. One patient developed multiple myeloma. The second patient who was later found to have a germline mutation in the BRCA 2 gene was diagnosed with pancreatic cancer. The third patient developed both a triple-negative (hormone receptor-negative and HER2-negative) breast cancer as well as rectal cancer. Of the healthy control subjects, one was diagnosed with ductal carcinoma in situ (DCIS) of the breast that was hormone receptor-positive.

### Gut microbiome results

**Fig. 1:**
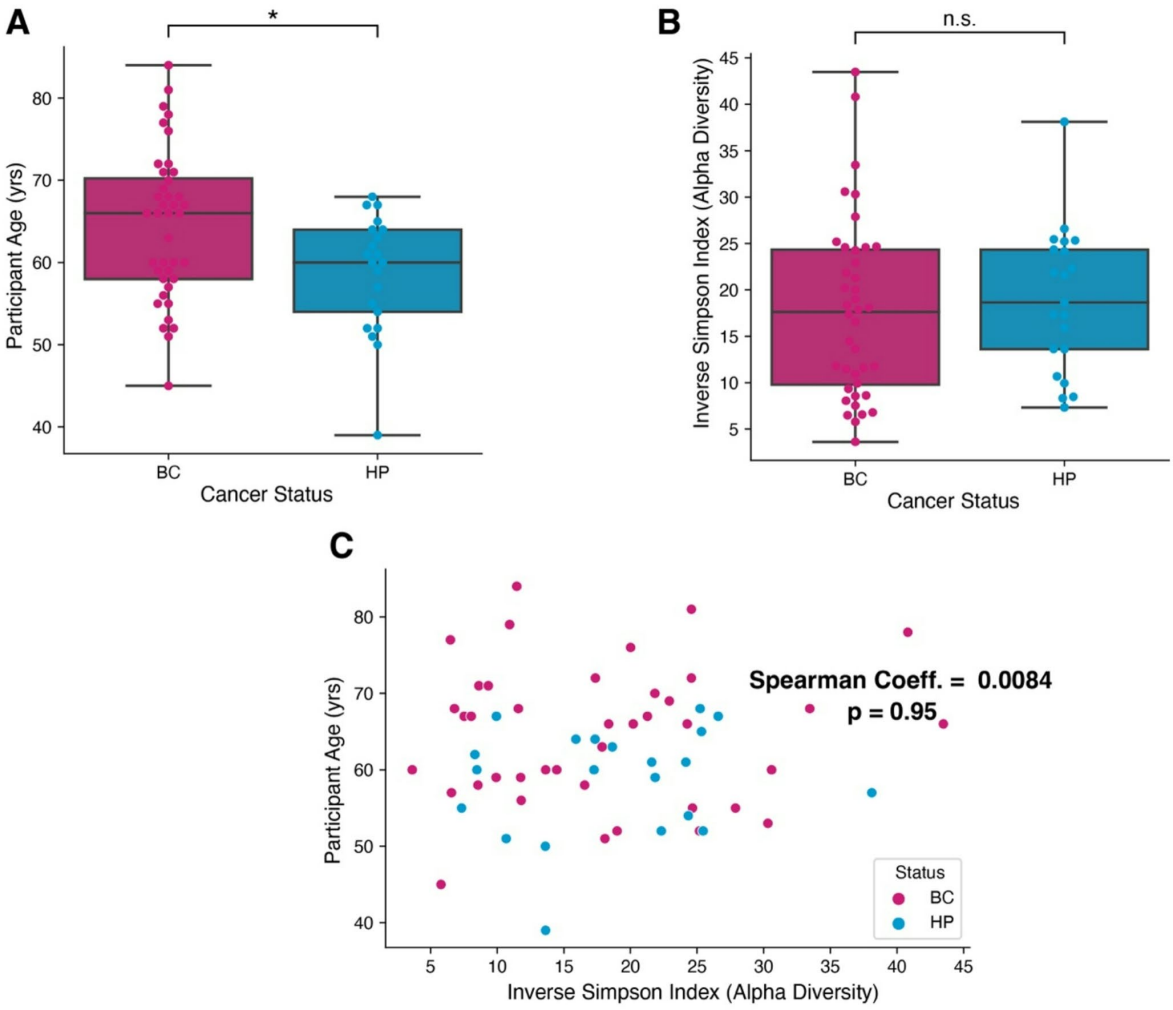
Alpha diversity and age across all participants. (**A**) Age and (**B**) alpha diversity in breast cancer patients and healthy study participants (HP) (*p* = 0.01) (**C**) Correlation between age and diversity (Spearman correlation coefficient = 0.00084; *p* = 0.95)

**Fig. 2:**
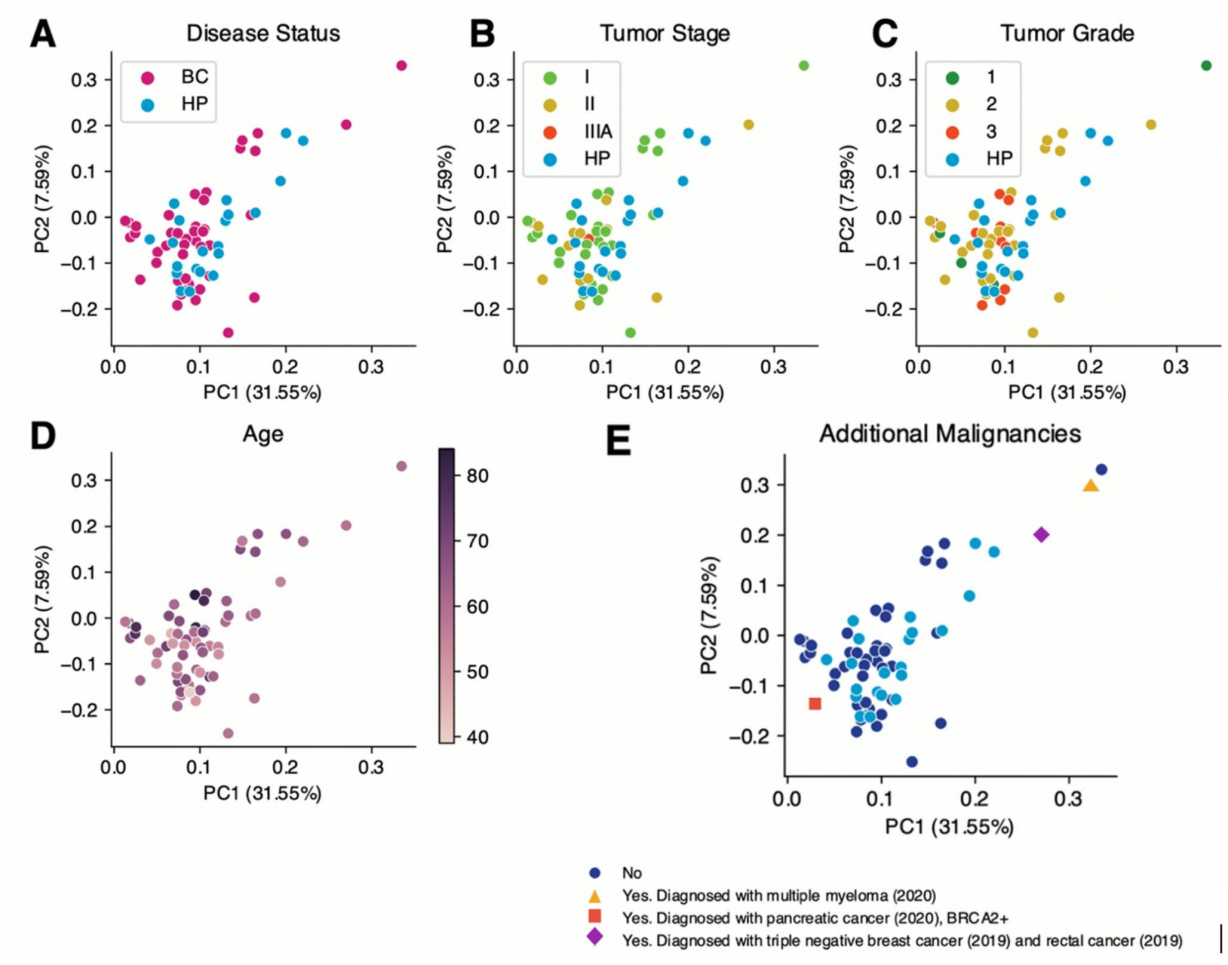
Principal component analysis of participants by disease status (**A**), tumor stage (**B**), tumor grade (**C**), age (**D**), and additional malignancies (**E**)

We first deduced whether the bacterial compositions of participant stool samples correlated with any common clinical confounders. Although age does differ between the HP/BC groups (*p* = 0.01, Fig. 1A), alpha diversity was not significantly different in the groups (*p* = 0.65, Fig. 1B) and age did not correlate with alpha diversity in our samples (Fig. 1C). In addition, we investigated whether there was a separation based on alpha diversity between tumor grade and cancer stage and found no significant differences between groups (Supplementary Figure 2). We then compared sample composition by principal coordinate analysis and found no clustering by our metadata variables (e.g., breast cancer vs healthy control, cancer stage, tumor grade, age) (Fig. 2). There was also not a significant difference in terms of BMI or probiotic use. Of note, the two breast cancer patients that were later diagnosed with myeloma and pancreatic cancer, respectively, were found in opposite extremes on the first principal component and separated from healthy or other breast cancer subjects (Fig. 2E, Supplementary Figure 3).

**Fig. 3:**
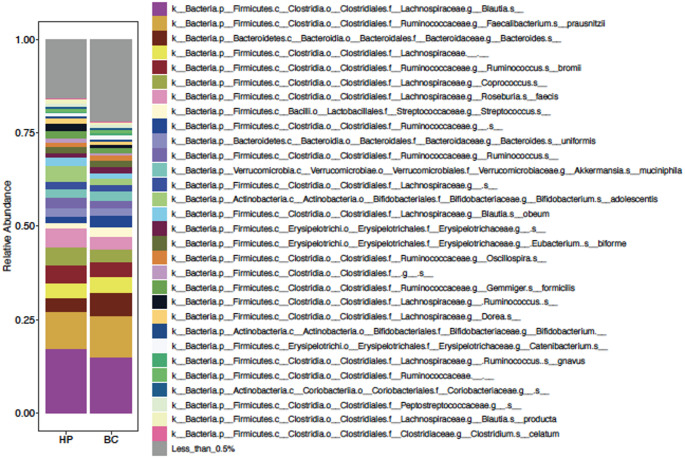
Taxa composition of breast cancer and healthy control cohorts. Breast cancer patients and healthy participant microbial composition

**Fig. 4:**
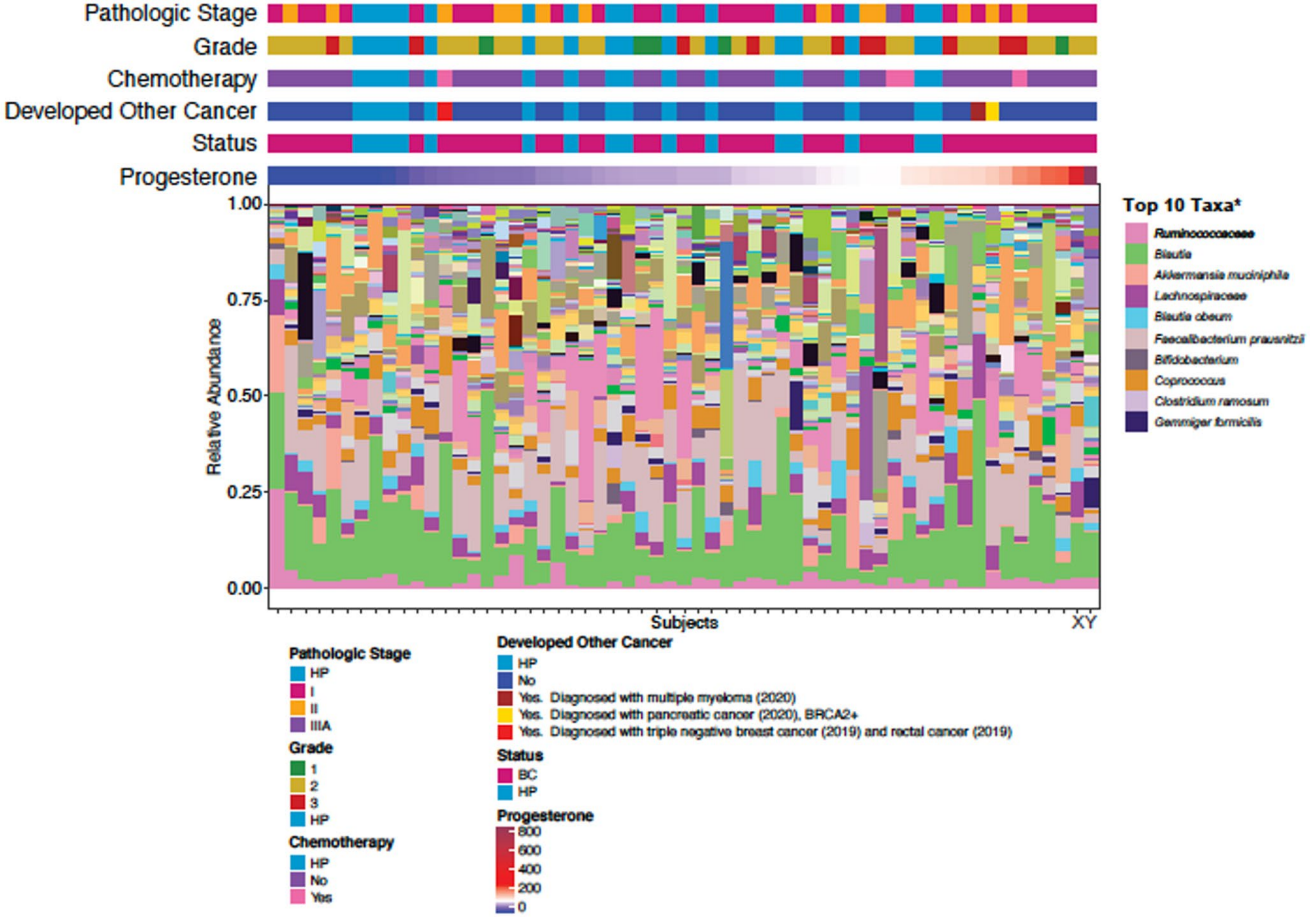
Relative abundance of taxa across creast cancer samples. *Additional taxa key available in Supplementary Fig. 4

**Fig. 5:**
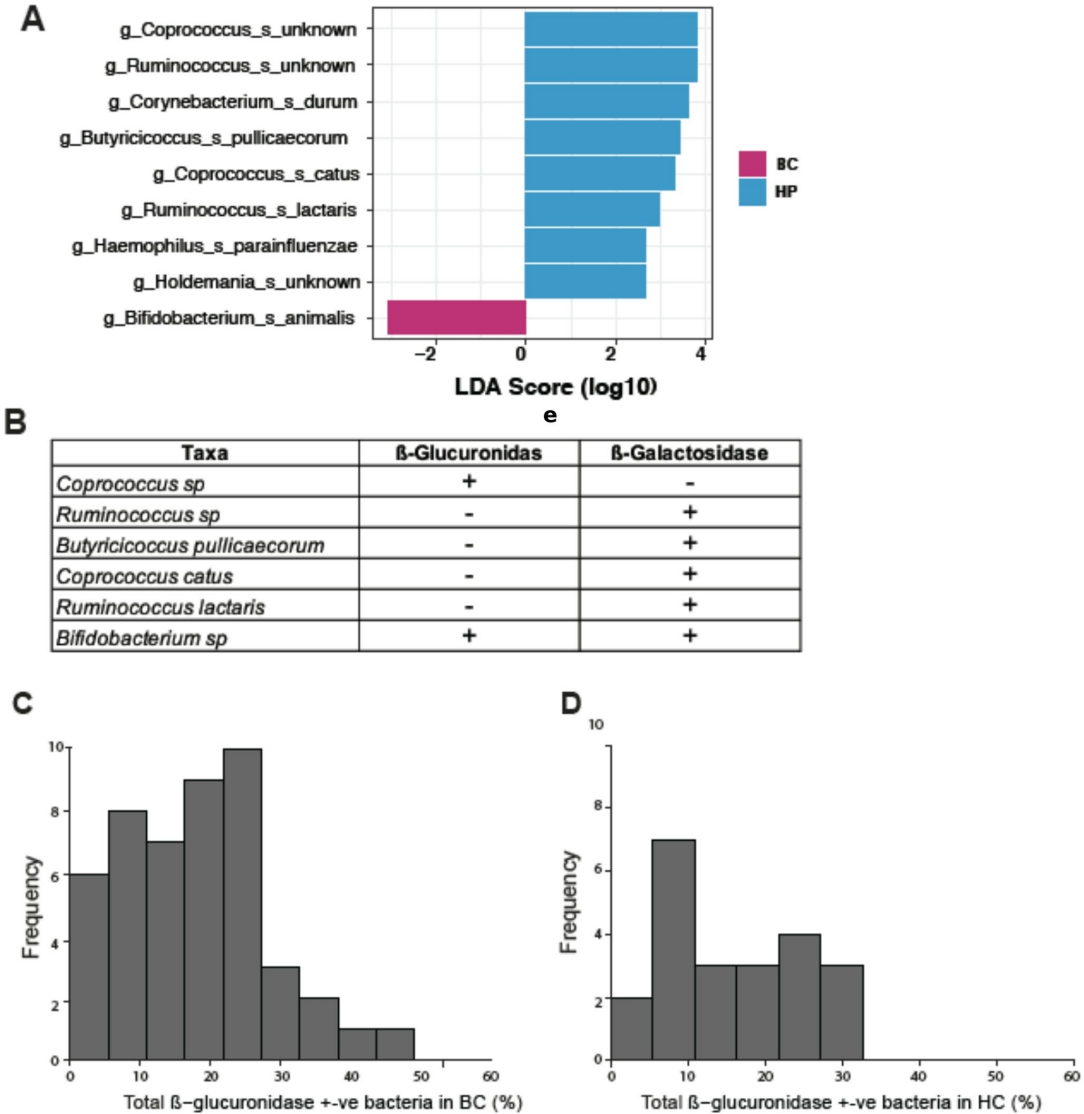
Differential taxa abundance (**A**) and distribution of selected beta glucuronidase positive (**B**) bacteria in cancer and control cohorts. Histograms show summed relative abundances of beta glucuronidase bacteria in a sample for breast cancer patients (**C**) and healthy controls (**D**)

We then examined the relative microbial abundance on the species level of the most abundant species (abundance > 0.5%) in the two groups (Figs. 3 and 4). To investigate whether these differences in taxa composition were related to differences in progesterone level (the only sex hormone found to be significantly different between cohorts, see section below), we looked at the relative taxa abundance in each sample sorted by progesterone level (Fig. 4). We identified a few samples dominated by one or two bacterial taxa (e.g., *Blautia* and *Ruminococcaceae*). We also observed altered microbial composition in samples from two breast cancer subjects with the highest progesterone levels in the urine (Fig. 4, Subjects X & Y). In order to identify microbes with statistically different abundance between breast cancer subjects and healthy controls, we used Linear discriminant analysis Effect Size analysis. This revealed that *Bifidobacterium animalis* was overrepresented in breast cancer patients, while eight species were found to have higher abundance in healthy controls (Fig. 5A). *B. animalis* can produce beta glucuronidase, and therefore contribute to the deconjugation of estrogen, leading to reabsorption in the circulation (Kwa et al. 2016).

We further visualized the abundance of these the nine taxa as box plots to compare their abundance between breast cancer subjects and healthy controls. This revealed that 4 of the 9 taxa identified in LDA were very low abundance (< 0.01% abundance for all samples), except for *Coprococcus (unknown species), Ruminococcus (unknown species), C. cactus, B. animalis,* and *H. parainfluenzae* (Supplementary Figure 5). Of note, the differences between the breast cancer subjects and healthy controls were statistically significant or near-statistically significant for *Coprococcus (unknown species)* and *Ruminococcus* (*unknown species*) abundance as reported by an independent two-tailed T-test (*p* = 0.0575 and *p* = 0.0225, respectively). When removing the breast cancer subjects who underwent additional chemotherapy treatment, both taxa became increasingly significant (*p* = 0.0165 and *p* = 0.0246, respectively).

To analyze the potential estrogen-modulatory capacity of the different microbial compositions across our samples, we combined the relative abundances of all bacteria reported to possess beta glucuronidase genes (Pollet et al. 2017). The distribution of beta glucuronidase positive bacteria in samples from breast cancer subjects was slightly skewed towards larger values compared with samples from the healthy controls (Fig. 5C and D, Supplementary Figure 6); however, this difference was not statistically significant. Interestingly, both, breast cancer and healthy subjects showed wide distributions of the prevalence of beta-glucuronidase positivity, reaching from 0 to 33% in healthy controls and 0 to 49% in breast cancer subjects (Fig. 5C and D). To account for this wide variability, we conducted a Bayesian analysis to estimate the difference in the average abundances of beta glucuronidase positive bacteria indicating 74% probability that breast cancer subjects have higher average beta glucuronidase levels (Supplementary Figure 6). Lastly, a study by Ervin et al., identified 13 additional bacteria that possess beta-glucuronidase positive genes, 5 of which were found in our data (Ervin et al. 2019). None of these species showed statistical significance between breast cancer patients and healthy participants was shown (Supplementary Figure 7).

**Fig. 6:**
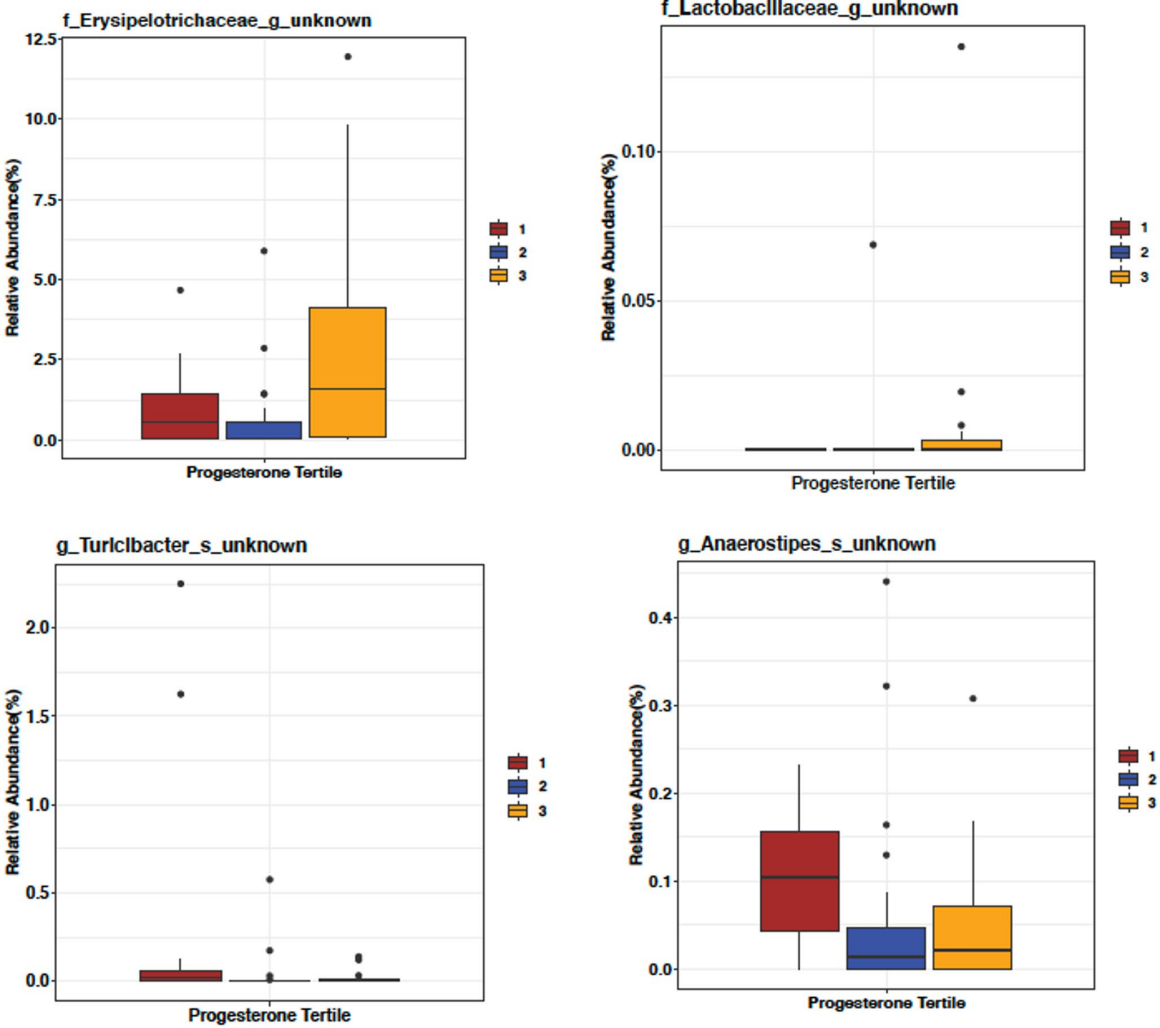
Altered taxa abundance by progesterone tertiles. Cancer samples were divided by progesterone group into tertiles (1-Low, 2-Medium, 3-High). Pairwise Kruskal–Wallis test was performed to compare the progesterone tertiles with each of the bacteria identified previously. Bacteria with a *p* value < 0.05 were selected for the post-hoc Mann–Whitney test between the progesterone tertiles with Benjamini–Hochberg p value adjustment. The reported bacteria had at least one comparison with a *p* value < 0.05

Finally, samples were split by progesterone tertiles to determine if any particular taxa were associated with progesterone levels. We identified four taxa (*Erysipelotrichaceae, Lactobacillaceae, Turicibacter* and *Anaerostipes*) with significantly altered abundance. Specifically, the highest progesterone tertile group had increased relative abundance of *Erysipelotrichaceae* and *Lactobacillaceae* and reduced relative abundance of *Turicibacter* and *Anaerostipes* (Fig. 6).

### Sex hormone results

The breast cancer subjects were found to have a significantly higher progesterone level in the urine (*p* = 0.036) compared to controls (Table 3). There was also a trend towards lower plasma progesterone in the breast cancer cases (*p* = 0.054) (Table 4). Analyses of plasma and urine estrogens (parent estrogens and metabolites) and testosterone levels did not reveal significant differences between both groups and this applied also to circulating SHBG.

**Table 3:** Urinary steroid excretion results

MEAN				
	Cases (pg/mg) *n* = 46	Controls (pg/mg) *n* = 22	Case/Control (%)	P*
E1	4.5	5.1	89%	0.57
E2	1.2	1.3	93%	0.76
2-OHE1	7.5	8.4	89%	0.71
2-OHE2	1.4	1.5	95%	0.85
4-OHE1	1.1	1.5	72%	0.30
16-OHE1	1.3	1.3	105%	0.86
16-ketoE2	0.7	3.3	21%	0.12
2-MeOE1	1.3	2.5	52%	0.17
E3	5.6	5.0	112%	0.67
Test	3.8	3.9	96%	0.82
Prog	0.2	0.1	181%	**0.036**

Estrone (E1), estradiol (E2), estriol (E3), 2-hydroxyestrone (2-OHE1), 2-hydroxyestradiol (2-OHE2), 2-methoxyestrone (2-MeOE1), 2-hydroxy-3-O-methylestrone (2OH-3MeO-E1), 4-hydroxyestrone (4-OHE1), 4-hydroxyestradiol (4-OHE2), 16α-hydroxyestrone (16α-OHE1), 16-ketoestradiol (16keto-E2), progesterone (Prog), and testosterone (Test). All urinary levels were adjusted to creatinine and expressed as pg per mg creatinine. *by unpaired t-test with common variance

**Table 4:** Plasma steroid and SHBG results

MEAN				
	Cases [stdev] *n* = 46	Controls [stdev] *n* = 22	Case/Control	P*
E1				
Total (pg/mL)	199 [177]	195 [139]	102%	0.92
Total/SHBG (pg/pmol)	6 [12]	4 [4]	144%	0.50
Unconjug (pg/mL)	11[(Polley et al.^2013^)]	10 [(Polley et al. 2013)]	103%	0.84
Unconjug/SHBG (pg/pmol)	0.23 [0.24]	0.21 [0.17]	106%	0.81
Free (pg/mL)	0.294 [0.193]	0.282 [0.156]	104%	0.81
E3				
Total (pg/mL)	15 [24]	17 [37]	87%	0.77
Total/SHBG (pg/pmol)	0.39 [0.99]	0.33 [0.54]	119%	0.78
Unconjug (pg/mL)	n/a	n/a		
Unconjug/SHBG (pg/pmol)	n/a	n/a		
Free (pg/mL)	n/a	n/a		
Test				
Total (pg/mL)	136 [112]	175 [175]	78%	0.27
Total/SHBG (pg/pmol)	3.2 [5.3]	3.4 [3.5]	94%	0.87
Unconjug (pg/mL)	39 [72]	44 [80]	88%	0.80
Unconjug/SHBG (pg/pmol)	1.0 [3.0]	0.9 [1.7]	114%	0.83
Free (pg/mL)	1.8 [2.1]	2.1 [2.2]	84%	0.54
Prog				
Total (pg/mL)	43 [44]	78 [108]	55%	0.054
Total/SHBG (pg/pmol)	0.9 [1.1]	1.6 [2.2]	56%	0.10
Unconjug (pg/mL)	33 [21]	51 [98]	65%	0.22
Unconjug/SHBG (pg/pmol)	0.7 [0.7]	1.1 [1.8]	63%	0.20
Free (pg/mL)	n/a	n/a		
SHBG (pmol/mL)	70 [42]	63 [37]	111%	0.50

Estrone (E1), estriol (E3), progesterone (Prog), testosterone (Test), SHBG (steroid hormone-binding globulin), standard deviation (stdev), unconjug (unconjugated). *by unpaired t-test with common variance

To probe if urine estrogen levels were correlated with the abundance of taxa, we conducted a correlation analysis of urine estrogen vs bacterial taxa. We identified six species with statistically significant correlations between urine estrogen and their abundance (Supplementary Figure 8). Of note, three of the significant hits had less than 10 samples containing nonzero species abundance (*Acidaminococcus (unknown sp), Desulfovibrio D168,* and *Lactobacillus helveticus*) suggesting the possibility of a spurious correlation. None of the significant bacteria species were reported as BG + . However, other members of the *Roseburia* and *Lactobacillius* genus do contain species that are BG + .

## Conclusions

A potentially important role of the gut microbiome (estrobolome) is the modulation of estrogen homeostasis through the enterohepatic circulation of estrogens, which may affect breast cancer risk. In this study, we investigated whether the pretreatment gut microbiome differed in postmenopausal patients with hormone receptor-positive breast cancer versus healthy subjects, while controlling for potential confounders and focusing upon bacterial taxa with β-glucuronidase activity. We evaluated gut microbiome diversity by analyzing how many different taxa are present, as well as how evenly distributed the taxa are amongst the two cohorts. When comparing the percentages of β-glucuronidase positive bacteria, there was no statistically significant difference, however there was evidence that some bacterial taxa with β-glucuronidase were enriched in the breast cancer patients. The breast cancer subjects had a higher abundance of *Faecalibactreium prausnitzi, Bacteroides* species, and *Bifidobacterium animalis,* which contain β-glucuronidase activity, whereas abundances of several taxa without β-glucuronidase activity were reduced, like *Blautia*, *Coprococcus*, *Roseburia faecis* and *Bifidobacterium adolescentis*. Interestingly, we also discovered a wide distribution of prevalence of β-glucuronidase positive taxa in both the breast cancer subjects and healthy controls. This suggests a wide range of potentially important effects of the gut microbiome’s metabolic capacity on estrogen metabolism and host physiology, and this finding warrants further investigation. In comparison, a pilot study investigating differences in the gut microbiome among postmenopausal women found a less diverse microbiome and significantly altered microbiota composition in newly diagnosed breast cancer patients prior to treatment compared with healthy controls (Goedert et al. 2015). Levels of systemic estrogens were also higher in the breast cancer patients, although these were independent of differences in the microbiome, suggesting that the gut microbiota may affect breast cancer risk through an estrogen-independent pathway.

In our study, the breast cancer subjects unexpectedly did not have significant differences in systemic estrogen levels compared to the healthy control subjects. The reason behind this is unclear, and may be a reflection of the small sample size or other variables in terms of sample recruitment. Intriguingly, endogenous progesterone levels were significantly higher in the urine of breast cancer patients compared to healthy controls, and there was a nonsignificant trend toward lower plasma levels in the patients. Estrogen and progesterone are both involved in breast development during puberty, primarily through a paracrine mechanism that leads to cellular proliferation in the mammary gland (Asselin-Labat et al. 2010). Epidemiologic studies have shown a well-established increase in breast cancer risk with use of exogenous progestogens (progestins) administered together with estrogen as menopausal hormone replacement therapy (Cancer 1997; Manson et al. 2013). In addition, breast cancer risk from the use of oral hormonal contraceptives may primarily be attributed to the usage of progestins (Niemeyer Hultstrand et al. 2022). Hormonal contraception may influence the gut microbiome through various mechanisms which may include alterations in microbial diversity and composition and changes in activity of the estrobolome (Mihajlovic et al. 2021). In contrast, the role of endogenous progesterone in breast cancer carcinogenesis is not as well defined, and fewer studies have assessed circulating progesterone levels and breast cancer risk. One study of postmenopausal women that included over three hundred breast cancer cases and six hundred matched controls did not demonstrate an association between progesterone levels and risk for breast cancer (Missmer et al. 2004). However, this data was limited by close to 30% of samples having undetectable levels per the assay used for analysis. Another study in premenopausal women found a modest reduction in breast cancer risk with higher circulating progesterone levels (Micheli et al. 2004). The low levels of circulating progesterone levels in postmenopausal women and inadequate sensitivity of some assays can pose a challenge for detection, although with advances in assay technology, the assay used in our study was adequately sensitive to detect a difference in progesterone levels. A more recent study showed an increase in breast cancer risk among postmenopausal women with higher endogenous levels of progesterone in the blood compared to lower levels (Trabert et al. 2020a, b). Whether progesterone levels are affected by the gut microbiome or vice versa is also unclear; we did observe an altered microbial composition in the fecal samples of two breast cancer subjects with the highest progesterone levels. Importantly, while progesterone assays capture progesterone levels in circulation at the time, they cannot assess the flux of progesterone, i.e., the rates of increase or decrease that are potentially affected by microbial metabolism in addition to homeostatic regulation. Progesterone may potentially influence the gut microbiome through multiple mechanisms, including effects on microbiome composition, immune modulation, and gastrointestinal physiology. Overall, our results suggest a potential role for endogenous progesterone in postmenopausal breast cancer risk; this and a potential modulating role of the microbiome warrants further study.

Limitations of our pilot study include its small sample size, potential for selection bias, and case–control design, which preclude the ability to demonstrate causation. Further use of shotgun metagenomics would allow for additional understanding of the role of additional taxa and specific gene families in breast cancer risk. Nonetheless, our findings provide an early step toward a better understanding of the role that bacteria with estrogen modulating activity may play in the risk of hormone-driven malignancies like breast cancer. Future important studies include evaluation of how glucuronidases from these taxa relate in sequence to the glucuronidases that process estrogen glucuronides as validated by Ervin et al. (Ervin et al. 2019), as well as how the active sites of these enzymes compare. We also plan to prospectively study the longitudinal role of estrogen deprivation during aromatase inhibitor therapy on the gut microbiome in our breast cancer subjects. We hope that our research will identify characteristics of the gut microbiome that could be used to develop novel and personalized approaches for breast prevention and treatment.

## Supplementary Information

Below is the link to the electronic supplementary material.

Supplementary Material 1

## Data Availability

No datasets were generated or analysed during the current study.
