# Supplementary material for: Evaluation of the gut microbiome and sex hormones in postmenopausal women with newly diagnosed hormone receptor-positive breast cancer versus healthy women: a prospective case-control study
Source: J Cancer Res Clin Oncol. 2025 Oct 4;151(10):275. doi: 10.1007/s00432-025-06338-z (PMC12494539; doi:10.1007/s00432-025-06338-z)
Supplement: Supplementary file 1 — Supplementary Material 1 [file 432_2025_6338_MOESM1_ESM.docx]

**Supplementary Material:**

**Baseline Questionnaire**

**A. MEASUREMENTS**

1. Standing height (cm)

|__|__|__|.|__|

2. Weight (kg)

|__|__|__|.|__|

**B. DEMOGRAPHICS**

1. Age

|__|__|__|.|__|

2. Which category best describes your race?

_____ Black or African American

_____ American Indian or Alaska Native

_____ White

_____ Asian/Pacific Islander

_____ Hispanic

_____ Other

**C. MEDICAL HISTORY**

1. Have you ever been told by a doctor, nurse, or other health care professional that you have anemia? Yes______ No______

2. Do you have any immune disorders? Yes______ No______

3. Do you have any bowel disorders or diarrhea? Yes______ No______

4. Have you had any stomach or bowel surgeries? Yes______ No______

5. Have you used any antibiotics over the past 6 months? Yes______ No______

6. Have you used any hormones (by mouth, patch or intravaginal) such as hormone replacement therapy (Setiadi et al.) over the past 12 months? Yes______ No______

7. Have you used any other medications over the past 2 months? Yes______ No______

8. If yes, indicate the use in the last 2 months:

| **Medication Type** | **Name** | **Dose** | **Date Started** | **Current Use (Yes/No)** |
| --- | --- | --- | --- | --- |
| Antibiotics | | | | |
| Pain killers | | | | |
| Antacids | | | | |
| Hormones | | | | |
| Steroids | | | | |
| Other | | | | |
| Other | | | | |

**D. CIGARETTE SMOKING HISTORY**

1. Do you currently smoke cigarettes? Yes______ No______

**E. DIETARY HISTORY**

1. Do you consider yourself as eating a ‘normal’ balanced diet? Yes______ No______

2. Do you use probiotics on a daily basis or in the past 6 months? Yes______ No______

3. Do you eat a vegetarian diet? Yes______ No______

4. Do you drink alcohol? Yes______ No______ If yes, how many glasses per week? _________

**Supplemental Figure 1. Baseline Patient Questionnaire**

**
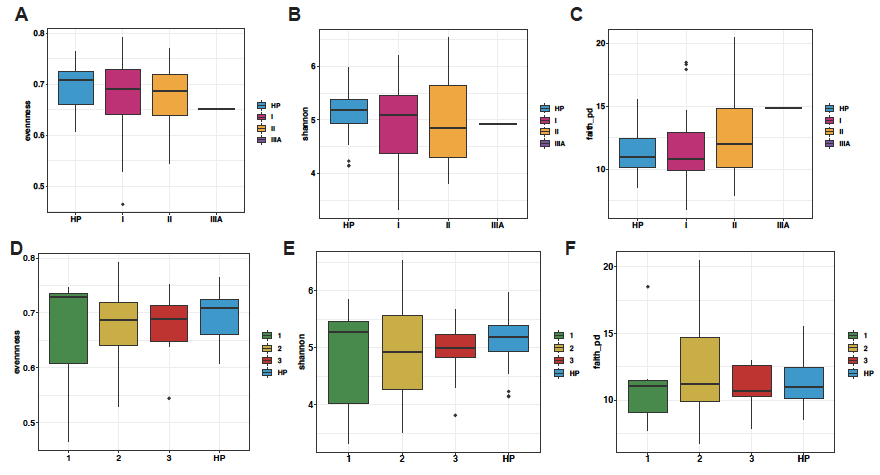
**

**Supplemental Figure 2. Alpha diversity of breast cancer and healthy control participants by stage and grade**

**
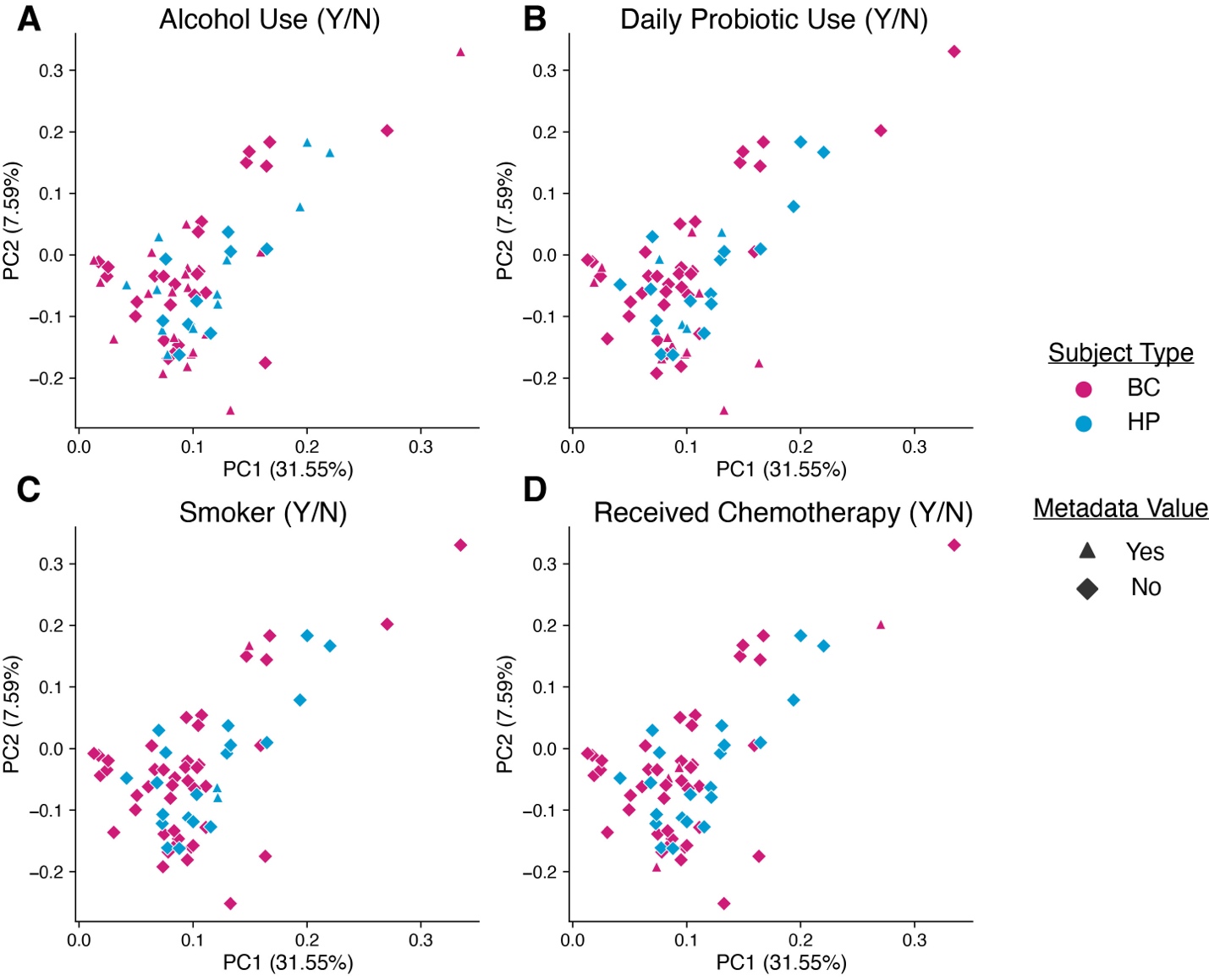
**

**Supplementary Figure 3. Beta diversity of breast cancer and healthy control participants** for alcohol use (A), probiotic use (B), smoking status (C) and chemotherapy (D).

**Supplementary Figure 4. Taxa key for Figure 4**


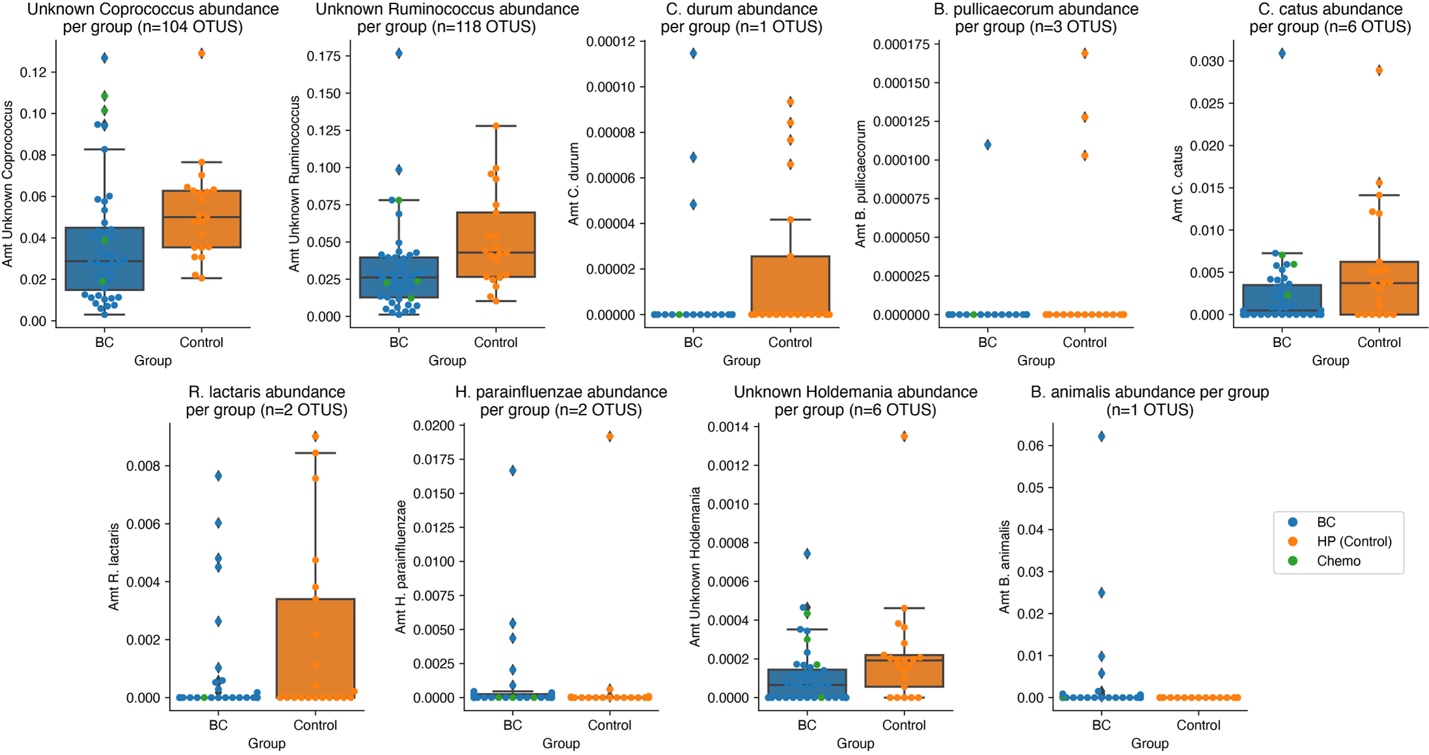


**Supplementary Figure 5. Relative abundances between BC and HC in the LDA-identified taxa.** Box plots were used to visualize abundance of taxa identified in the LDA screen as significantly different in HP and BC subjects in orange and blue, respectively. Green represents the samples of patients who underwent further chemotherapy treatment.


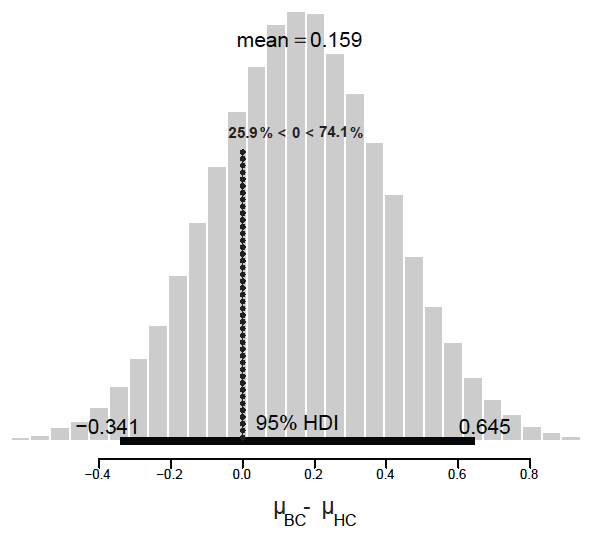


**Supplementary Figure 6.** **Bayesian analysis of difference in mean beta glucuronidase bacteria relative abundances between BC and HC.** The posterior distribution of estimated differences in the means of standardized (z-score) beta glucuronidase relative abundances is shown, and the 95% highest density interval (HDI) is indicated; the positive and negative posterior densities are annotated with a mean difference of 0.159 standard deviations estimated. The dotted line indicates a difference in means of 0.


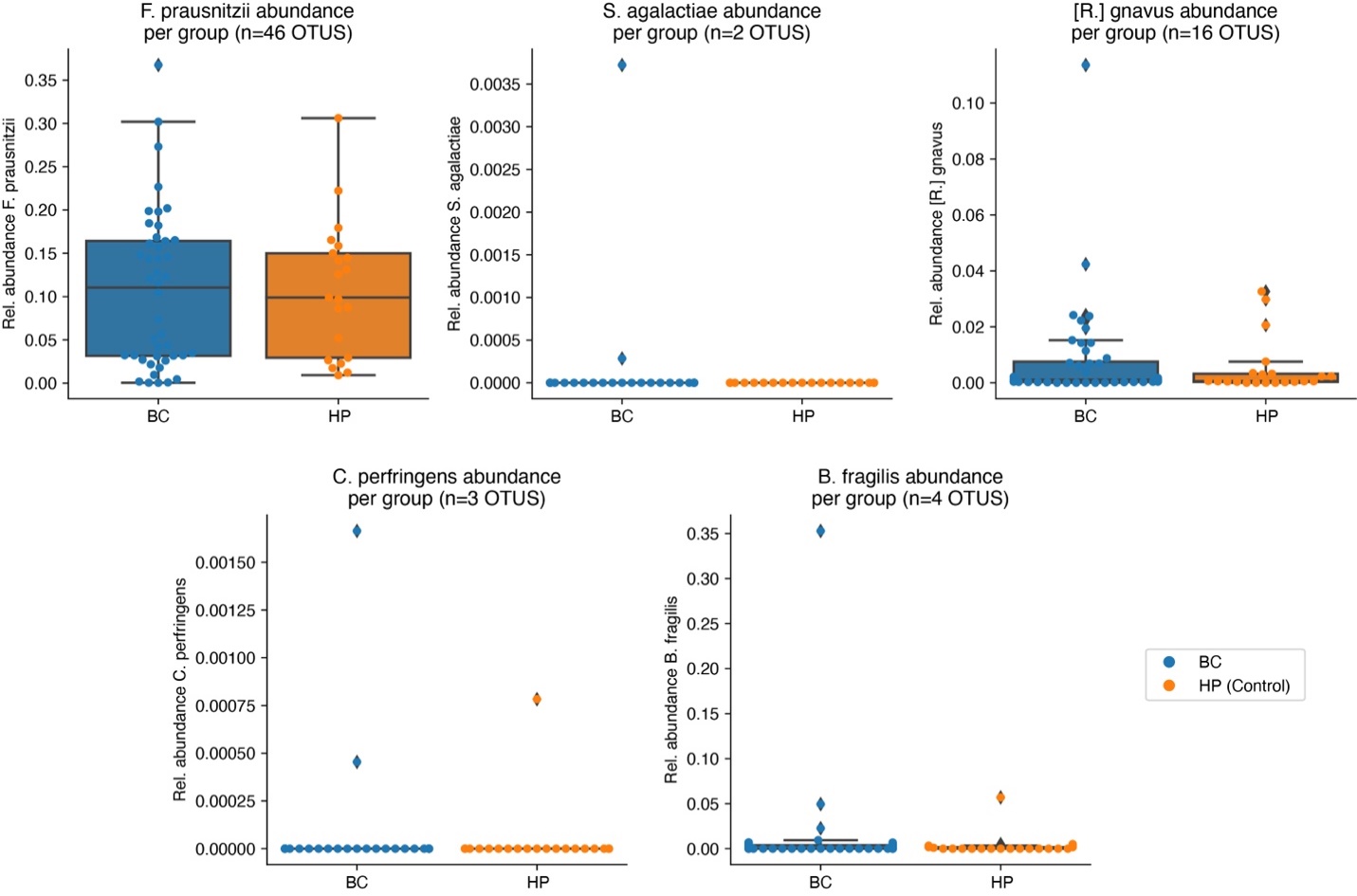


**Supplementary Figure 7. Additional β-glucuronidase-positive species found in Ervin, et al. 2019 JBC do not show significant difference between BC and healthy controls.** An additional paper described 13 additional bacteria species that contain β-glucuronidase-positive genes. Only 5 of these species showed nonzero abundance in our data, and none had a significant p-value as reported by t-test (p=0.96, 0.46, 0.5, 0.86, and 0.51 for *F. prausnitzii, S. agalactiae, [R.] gnavus, C. perfringens,* and *B. fragilis*, respectively).


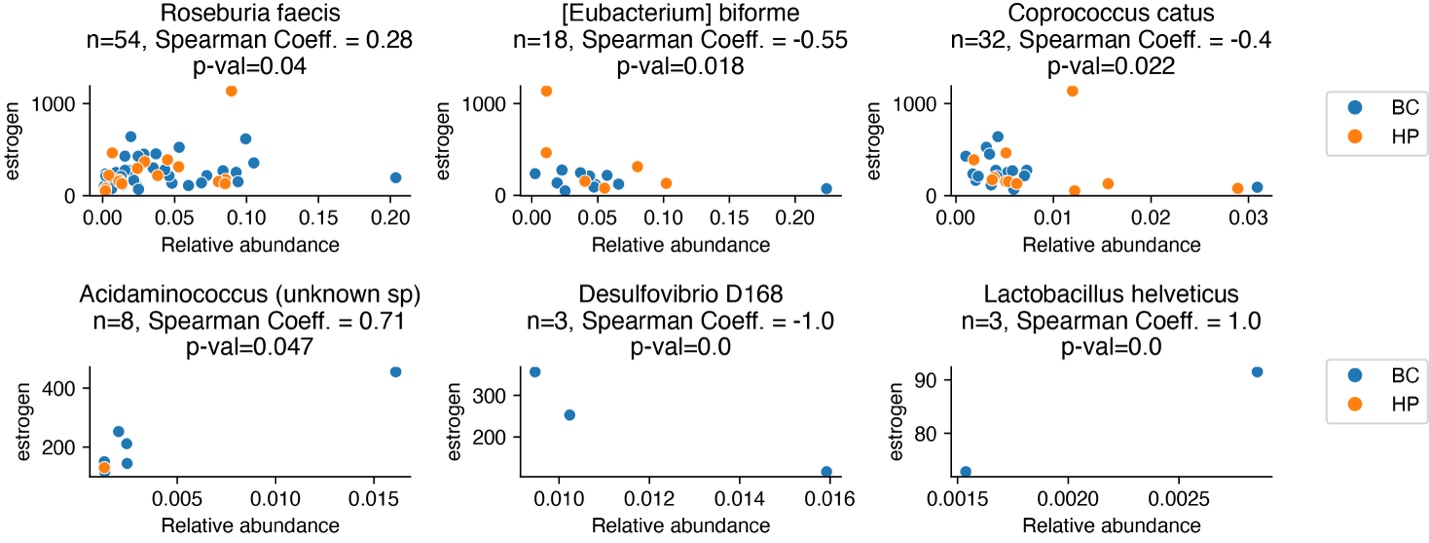


**Supplementary Figure 8. Taxonomy-wide Spearman correlation between urine estrogen and relative abundance yields six significant results.** Notably, *Acidaminococcus (unkown sp), Desulfovibrio D168,* and *Lactobacillus helveticus* have less than 10 samples with nonzero series abundance.
